# Prevalence and determinants of adverse pregnancy outcomes among teenage mothers in the Limbe Health District of Cameroon

**DOI:** 10.11604/pamj.2025.51.5.43080

**Published:** 2025-05-07

**Authors:** Ndip Esther Ndip, Nana Njamen Theophile, Chrisantus Eweh Ukah, Claudia Ngeha Ngu, Mirabelle Pandong Feguem, Randolf Fuanghene Wefuan, Larissa Kumenyuy Yunika, Syveline Zuh Dang, Egbe Obinchemti Thomas

**Affiliations:** 1 Department of Public Health and Hygiene, Faculty of Health Sciences, University of Buea, P.O. Box 12, Buea, Cameroon,; 2 Department of Obstetrics and Gynecology, Faculty of Health Sciences, University of Buea, P.O. Box 12, Buea, Cameroon

**Keywords:** Teenage pregnancy outcomes, determinants, prevalence, Limbe Health District

## Abstract

**Introduction:**

teenage pregnancy constitutes a serious health and social problem worldwide. World estimates report about 16 million births to adolescent mothers, most of them occurring in low and middle-income countries often accompanied with adverse outcomes. Cameroon's adolescent fertility rate of 138 births per 1000 women aged < 19 is the highest in Central Africa. This study aimed to determine the prevalence and determinants of adverse teenage pregnancy outcomes among adolescents in the Limbe Health District.

**Methods:**

cross-sectional study was conducted among 220 participants from November 2022 to May 2023. A multistage sampling technique was used to recruit participants. Descriptive statistics and logistic regression were used to summarize the data and identify factors associated with adverse teenage pregnancy outcomes respectively. Statistical significance was set at p<0.05.

**Results:**

the findings showed that the prevalence of adverse teenage pregnancy outcomes was 34.8%, with caesarean delivery (20.9%), preterm delivery (12.7%), low birth weight (19.5%), and neonatal mortality (9.1%) being the main adverse outcomes of teenage pregnancy. The determinates of caesarean delivery were drug use (AOR= 5.02, 95% CI: 1.98-12.71), premature delivery (AOR= 13.19, 95% CI: 2,25-77.25), and physical exercise (AOR= 0.16, 95% CI: 0.06-0.45), for premature delivery were age group 17-19 years (AOR= 0.20, 95% CI: 0.07-0.60) and smoking (AOR= 6.93, 95% CI: 1.57-30.60), for low birth weight, were age group (AOR= 0.17, 95% CI: 0.08-0.39), delivered prematurely (AOR= 2.46, 95% CI: 1.11-5.43), and followed medical prescription (AOR= 0.21, 95% CI: 0.09-0.46) and finally for neonatal mortality were residence (AOR= 0.32, 95% CI: 0.16-0.67) and those who were properly fed (AOR= 6.61, 95% CI: 1.26-34.69).

**Conclusion:**

there is a high prevalence of adverse pregnancy outcomes among teenage mothers in the Limbe Health District. Awareness on pregnancy prevention needs to be raised among this population in order to reduce the burden of teenage pregnancy and its associated consequences on both the mother and the child.

## Introduction

Adolescent pregnancy remains a significant public health challenge globally, with profound implications for the health and well-being of young mothers and their children [1]. According to the World Health Organization (WHO), approximately 16 million girls aged 15 to 19 give birth each year, with the majority of these births occurring in low- and middle-income countries [2]. In Cameroon, the prevalence of adolescent pregnancies is alarmingly high, with estimates indicating that about 56% of young women in this age group have experienced pregnancy at least once [1]. The Limbe Health District, located in the Southwest Region of Cameroon, is no exception, facing a rising trend in teenage pregnancies that raises concerns about maternal and neonatal health outcomes. Adverse pregnancy outcomes, including preterm birth, low birth weight, and maternal complications, are disproportionately high among teenage mothers [3]. These outcomes can be attributed to various factors, including socioeconomic status, educational attainment, access to healthcare services, and cultural attitudes toward adolescent motherhood.

Teenage mothers often face barriers to accessing prenatal care, which can lead to inadequate monitoring of their health and that of their unborn child [4]. Additionally, they may lack the necessary knowledge and skills to navigate pregnancy and childbirth effectively, further increasing their risk of experiencing adverse outcomes. In the Limbe Health District, limited research has been conducted to explore the prevalence and determinants of adverse pregnancy outcomes among teenage mothers. Understanding these factors is crucial for developing targeted interventions aimed at improving maternal and child health in this population. Identifying the specific determinants that contribute to adverse outcomes can inform policymakers and healthcare providers in designing effective programs that address the unique needs of teenage mothers. This study aims to assess the prevalence of adverse pregnancy outcomes among teenage mothers in the Limbe Health District and to identify the key determinants influencing these outcomes. By shedding light on this critical public health issue, we hope to contribute valuable insights that will guide future interventions and improve health outcomes for young mothers and their children in Cameroon.

## Methods

**Study design and setting:** this study was a community-based cross-sectional study carried out in selected communities in the Limbe Health District, Cameroon from November 2022 to May 2023. The Limbe Health District is located in the South West Region of Cameroon. It has many recreational sites like the Botanic Garden and Seme Beach. It is one of the safest districts within the two English speaking Regions of Cameroon affected by the crisis.

The study population consisted of teenage girls between the ages of 10-19 residing in the Limbe Health District who were present at the time of the study. Teenage girls who had been pregnant and gave their consent were included in the study. Those who were severely ill or who had cognitive impairment were also excluded from the study. The sample size calculation was based on the Lorentz formula of sample size determination for prevalence study.


$$n=\frac{Z^{2}p\left(1-p\right)}{d^{2}}$$

Where, n is the minimum required sample size, Z is the standard normal deviate at 5% level of significance (1.96), p is the estimated proportion of the population, q = (1-p) and α is the precision of the estimate (0.05). A pre-estimate value of P= 13.3% was used. This was in accordance with a similar study in South West-Cameroon where the prevalence of teenage birth was 13.3% [5].

n=[(1.96)^2^ 0.133(1-0.133))/(0.05)^^2^^]

Where n= 178 participants, we decided to make up the sample size to 220 participants. A multi-stage random sampling method was used in this study. A simple random sampling technique was used to select 3 of the 8 health areas (Batoke, Bojongo, Bota, Idenau, Mabeta, Moliwe, Seaport, and Zone II) of the Limbe Health District. A probability proportionate technique size was used to know the number of participants to select from each health area to meet up the sample size of 220. Consecutive sampling was then used to recruit teenage mother to make up the sample size.

**Data collection:** data was collected using an interviewer-administered pretested structured questionnaire. The questionnaires were structured into three sections: sociodemographic, adverse outcomes of teenage pregnancy and determinants of adverse teenage pregnancy outcome assessment. The questionnaire was pre-tested in the Tole Health Area to ensure it was applicable to the study participants. The questionnaire was self-administered by teenage mothers who could read and write and by the data collector in cases where the participants could not read and write.

**Data management and analysis:** the questionnaires were cross-checked for completeness during data collection to limit missing data. Questionnaires with missing data were discarded. The data was keyed in to Epi Info version 7.2.4.0 statistical software and an Excel file was generated and the end of data entry and exported into Statistical Package for Social Sciences (SPSS) version 25 for analysis. Univariable analysis was done by descriptive statistics where continuous variables were summarized using means and standard deviations and categorical variables were described by using bar charts and tables. Simple logistic regression was used in the bivariable analysis since the response variables were binary, only variables that had significance of <0.2 was included into the multivariable analysis. In the multivariable analysis, multiple logistic regression analysis was done to determine the factors significantly associated with the adverse teenage pregnancy outcomes. Odds ratios (ORs) and their 95% confidence intervals (CIs) were calculated and a p value of <0.05 was considered statistically significant.

**Ethical and administrative approval:** ethical clearance to carry out this study was obtained from the Institutional Review Board of the Faculty of Health Sciences of the University of Buea. Administrative clearance was obtained from the Regional Delegation of Public Health for the South West Region of Cameroon, the Chief of service at the Limbe Health District and the chiefs of the community.

## Results

**Socio-demographic characteristics of the study participants:** regarding the socio-demographic characteristics of 220 participants (Table 1), their mean age was 17.6±1.4. A total of 170 (77.3%) were within the age group 17-19 and most 198 (90.0%) were single. Above half 118 (53.6%) lived in urban areas and 154 (70.0%) had attended just secondary education. A vast majority 186 (84.5%) were Christians and 120 (54.5%) were from a household size of 4-7. A total of 158 (71.8%) were unemployed and 110 (50.0%) did not have any monthly income. A total of 140 (63.6%) were living with their parents and about half 112 (50.9%) had not used drugs. Most 164 (74.5%) had not been sexually abused in the past and 116 (52.7%) had used contraceptives. Less than half 98 (44.5%) parental income was less than 50,000 and 202 (91.8%) gave birth in the hospital.

**Table 1:** socio-demographic characteristics of participants

Variable	Category	Frequency(n)	Percentage(%)
**Age group (Years)**	14-16	50	22.7
	17-19	170	77.3
	**Total**	**220**	**100**
**Marital status**	Single	198	90.0
	Married	22	10.0
	**Total**	**220**	**100**
**Residence**	Rural	102	46.4
	Urban	118	53.6
	**Total**	**220**	**100**
**Education**	No formal	2	0.9
	Primary	40	18.2
	Secondary	154	70.0
	Tertiary	24	10.9
	**Total**	**220**	**100**
**Religion**	Christian	186	84.5
	Muslim	34	15.5
	**Total**	**220**	**100**
**Household size**	1-3	72	32.7
	4-7	120	54.5
	8-10	28	12.7
	**Total**	**220**	**100**
**Occupation**	Unemployed	158	71.8
	Self-employed	56	25.5
	Government/Private employed	6	2.7
	**Total**	**220**	**100**
**Income**	None	110	50.0
**FCFA(XAF)**	<50000	102	46.4
	>50000	8	3.6
	**Total**	**220**	**100**
**Drug use**	No	112	50.9
	Yes	108	49.1
	**Total**	**220**	**100**
**Sexually abused**	No	164	74.5
	Yes	56	25.5
	**Total**	**220**	**100**
**Contraceptive use**	No	104	47.3
	Yes	116	52.7
	**Total**	**220**	**100**
**Place of delivery**	Not Hospital	18	8.2
	Hospital	202	91.8
	**Total**	**220**	**100**

**Figure 1:**
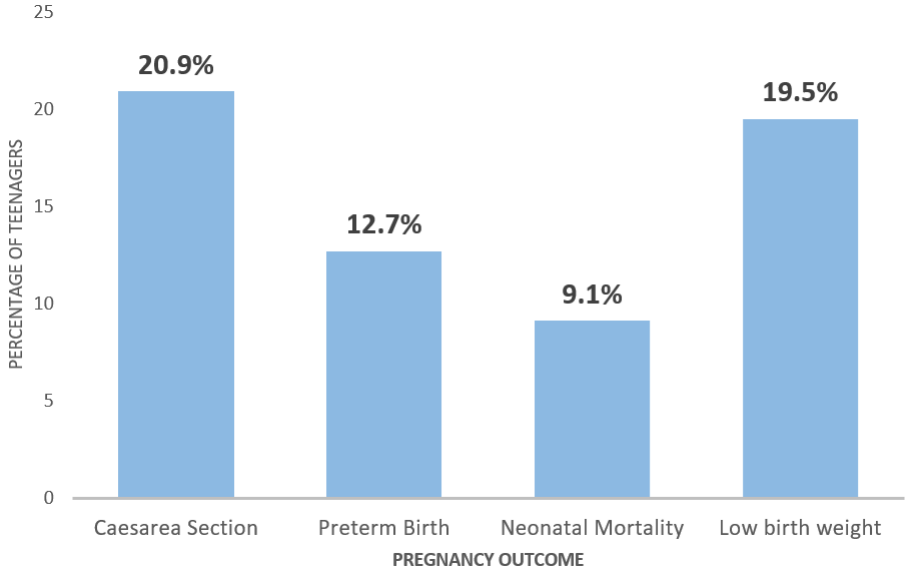
adverse maternal and neonatal outcomes of teenage pregnancy and their prevalence

**Prevalence of adverse teenage pregnancy outcomes:** the prevalence of adverse pregnancy outcome among teenagers was 34.80%. Figure 1 represents the main outcomes of teenage pregnancy, which were caesarean section (CS) delivery (20.90%), preterm birth (12.70%), neonatal mortality (9.10%) and low birth weight (19.54%). CS delivery was the most common adverse outcome (Figure 1).

**Determinants of adverse teenage pregnancy outcomes: caesarean delivery, premature delivery, low birth weight, and neonatal mortality:** Table 2 represents factors associated with CS delivery at the level of multivariable analysis. Factors finally found associated with CS delivery were drug use, premature delivery and physical exercise. A teenager using drugs during pregnancy was more likely to experience a CS (aOR: 5.02, 95% CI: 1.98-12.71; p=0.001) than those who were not using drugs. Those whose delivery was premature were more likely to deliver through CS (aOR: 13.19, 95%CI: 2.25-77.25; p=0.004) as compared to those who delivery was not premature. Teenagers who reported regularly engaged in physical exercise were less likely to deliver through CS (aOR: 0.16, 95%CI: 0.06-0.45; p=0.001). As shown on Table 3, age group and smoking status were factors found associated with premature delivery. Teenagers within the age group 17-19 years were less likely to deliver prematurely as compared to their younger counterparts within the age group 16-16 years (aOR: 0.20,95%CI: 0.07-0.60; P=0.011). Teenagers who were smoking while pregnant were about seven times more likely to deliver prematurely (aOR: 6.90, 95%CI: 1.57-30.60; p=0.011) as compared to those who were not smoking.

**Table 2:** factors significantly associated with caesarean delivery

	Delivered through CS		95CI for AOR		
**Variable**	**Category**	**AOR**	**Lower**	**Upper**	**p-value**
Age group (years)	17-19	0.552	0.226	1.349	0.193
	14-16	1	-	-	-
Drug use	Yes	5.022	1.984	12.708	**0.001**
	No	1	-	-	**-**
Premature delivery	Yes	13.189	2.252	77.248	**0.004**
	No	1	-	-	**-**
Physical exercise	Yes	0.159	0.056	0.451	**0.001**
	No	1	-	-	-

AOR=Adjusted Odd Ratio. CI=Confidence Interval, CS=Caesarean section

**Table 3:** factors significantly associated with premature delivery

			95% CI for AOR		
**Variable**	**Category**	**AOR**	**Lower**	**Upper**	**p-value**
Age group (years)	17-19	0.203	0.068	0.601	**0.004**
	14-16	1	-	-	-
Sexually abused	Yes	1.849	0.833	4.102	0.131
	No	1	-	-	-
Smoker	Yes	6.929	1.569	30.595	**0.011**
	No	1	-	-	-
Had a medical condition	Yes	0.812	0.368	1.794	0.607
	No	1	-	-	-

AOR=Adjusted Odd Ratio. CI=Confidence Interval

Table 4 represents factors independently associated with low birth weight. Four factors were finally identified as significant determinants of underweight baby delivery. These factors were age group, premature delivery, following medical prescription and maternal complication. The odds of a teenager within the age group 17-19 delivering an underweight baby were low (aOR: 0.17, 95%CI: 0.08-0.37; p<0.001) as compared to those within the age group 14-16 years. A baby delivered prematurely was more likely to be underweight (aOR: 2.5, 95%CI: 1.11-5.43; p=0.027). A teenage mother who followed all medical prescription was less likely to deliver an underweight baby (aOR: 0.2, 95%CI: 0.01-0.46; p<0.001) as compared to those who did not.

**Table 4:** factors significantly associated with low birth weight (multivariate analysis)

Variable	Category	AOR	Lower	Upper	p-value
Age group	17-19	0.172	0.076	0.386	**0.000**
	14-16	1	-		-
Used drugs	Yes	1.548	0.771	3.108	0.219
	No	1	-	-	-
Delivered prematurely	Yes	2.456	1.110	5.434	**0.027**
	No	1	-	-	**-**
Followed medical prescription	Yes	0.208	0.093	0.463	**0.000**
	No	1	-	-	-
Attended ANC	Yes	0.246	0.040	1.502	0.129
	No	1	-	-	-

AOR=Adjusted Odd Ratio. CI=Confidence Interval

Three factors were found associated with neonatal mortality as shown in Table 5. These factors were residence, feeding during pregnancy and child complication. The odds of a child born in urban area dying were 70% less than that of a child born in rural area (aOR: 0.3, 95%CI: 0.16-0.67; p=0.002). The odds of a child born of a woman who did not properly feed well during pregnancy dying were about seven times more than that of a woman who was properly fed (aOR: 6.60, 95%CI: 1.26-34.69; p=0.026).

**Table 5:** factors significantly associated to neonatal mortality

Variable	Category	AOR	Lower	Upper	p-value
Residence	Urban	0.323	0.156	0.672	**0.002**
	Rural	1	-	-	-
Consumed excess alcohol	Yes	3.273	0.899	11.915	0.072
	No	1	-	-	-
Physical exercise	Yes	2.718	0.903	8.182	0.075
	No	1	-	-	-
Properly fed	No	6.611	1.260	34.687	**0.026**
	Yes	1	-	-	-
Smoker	Yes	2.066	0.797	5.354	0.135
	No	1	-	-	-

AOR=Adjusted Odd Ratio. CI=Confidence Interval

## Discussion

This study aimed to assess the outcomes and determinants of adverse teenage pregnancy outcomes in both mother and child. The prevalence of adverse pregnancy outcome was 34.8%. This is similar to the 34.9% reported by Mezmur *et al*. (2021) [6] and higher than the 19% reported by Shri *et al*. in 2023 [7]. This high prevalence could be due to stigmatization which made it difficult for them to seek medical care and support [7,8]. This finding implies that targeted interventions and support services are urgently needed to address the health needs of pregnant teenagers.

For the main adverse pregnancy outcome, the proportion of teenage mothers who delivered though CS was 20.9%. This is similar to the 21.4% [9] reported in Ethiopia by Yisma *et al*. in 2019. This is however lower than the 36.8% proportion presented by Maskey in 2019 in a tertiary care hospital [10]. These results showed that a high proportion of teenagers are at risk of giving birth through CS and could result in further birth complications such as maternal and neonatal mortality if not properly managed. This 20.9% reported in this study may be because the study was carried out in a hospital and most of the teenagers who came were suspected to have health complications [11]. The proportion of teenagers who gave birth prematurely is 12.7%. This proportion is higher than the 6.2% reported by Hernandez in 2017 [12].

The proportion of babies with underweight was 19.38%. This proportion was lower than the 38.39% proportion reported by Tshotetsi in 2019 [13]. The 19.38% was higher than the 3.35% reported by Shen in 2019 [14]. This could be because of early gestational age and very poor feeding habits due to poverty. The proportion of neonatal mortality was 9.1%. The 9.1% is similar to the 15.61% reported by Chaibva in 2019 [15]. It was also lower than 34.7% reported by Weddih *et al*. in 2019 [16]. From our findings, the prevalence of neonatal mortality was very high. This could be due to poor prenatal care, and poor standard of living. The loss of a new-born can be emotionally devastating for parents and family members, and can lead to long-term psychological trauma [17]. Neonatal mortality can also lead to a reduction in human capital, as children who would have grown up to contribute to society are lost.

Regarding the determinants of adverse pregnancy outcome on the mother, factors found independently associated were:

***drug use:*** those who took drugs were more likely to deliver through CS. Drug use during pregnancy can increase the risk of complications during delivery; including the need for a caesarean and is associated with considerable obstetrical morbidity and mortality [18]. Nagandla in 2017 reported that drug dependency influenced the rate of caesarean section [19].

***Premature delivery:*** a teenage mother who gave birth prematurely was more likely to be born through CS than those not born prematurely. One reason why premature delivery may increase the likelihood of a CS delivery is that premature babies may be smaller and less developed than full-term babies [5]. This can make vaginal delivery more difficult and increase the risk of complications such as fetal distress, which may require an emergency CS delivery [20]. Premature delivery can also increase the risk of certain complications that may require a CS delivery [6].

***Physical exercise:*** teenagers who do exercise during pregnancy are less likely to deliver through CS than those who don't do exercise. Physical exercise during pregnancy may be associated with a reduced risk of CS delivery [20]. Regular exercise during pregnancy has been shown to improve maternal fitness, reduce the risk of gestational diabetes, and improve overall pregnancy outcomes.

***Age group:*** those between the ages of 15-19 were 0.23 times less likely to deliver prematurely than those who were below 15. This result from our study shows that age of maturity influences the delivery prematurely positively. A study by Akseer *et al*. reported a significant association between maternal age and premature delivery. One of the main reasons is that their bodies are not fully developed, which can lead to complications during pregnancy [21].

***Smoking:*** a teenager who smokes was more likely to delivery prematurely than those who did not smoke. Smoking is a well-known risk factor for premature delivery. When a pregnant woman smokes, the chemicals in the cigarette smoke can cross the placenta and reach the developing foetus [22]. This can cause a number of problems, including restricted growth, low birth weight, and premature delivery. Inflammation caused by the smoking can lead to damage to the placenta and lead to premature delivery [23].

For the determinants of adverse neonatal outcome of teenage pregnancy, factors found independently associated were:

***Age group:*** a teenager between the ages of 17-19 was less likely to have a baby that is underweight than those below 17 years. These babies born from younger mothers and are underweight, are at a higher risk of developing health problems both in the short-term and long-term. A study in 2022 reported that gestational age is a powerful predictor of birth weight and perinatal survival [24].

***Premature delivery:*** a teenager who delivered prematurely is 2.5 times more likely to have a baby with low birth weight. Premature babies are born before they have fully developed, which can result in a range of health problems such as low birth weight is associated with an increased risk of respiratory distress syndrome, infections, and other medical complications [25,26].

***Followed medical prescription:*** a teenager who followed medical prescription was less likely to have premature delivery. Medical prescriptions, such as prenatal vitamins, iron supplements, and medications to manage health conditions, are important for ensuring that both the mother and baby receive the nutrients and care they need to grow and develop properly [27].

***Residence:*** children born in rural areas were more likely to die as compared to those born in urban areas. Studies have shown that neonatal mortality rates are higher in rural areas compared to urban areas [28]. This is because mothers who live in rural areas may not have access to prenatal care, which can increase the risk of complications during pregnancy and childbirth.

***Proper feeding:*** a baby from a teenage mother who did not feed well is 6 times more likely to die than those who feed well. Poor feeding is a major contributor to neonatal mortality. Neonates are particularly vulnerable to malnutrition due to their small size and immature immune systems. Inadequate feeding can lead to a range of health problems, including low birth weight, hypoglycemia, hypothermia, and infections [29,30].

As strength, probability sampling was used which made the study externally valid as it gave all participants equal chances of being selected. For the limitation of the study, there might be recall bias due to participants' difficulty in accurately remembering events or experiences related to their teenage pregnancy outcome.

## Conclusion

There is a high prevalence of adverse teenage pregnancy outcomes which can place a significant economic burden on the society, through increased healthcare costs, lost productivity, and increased social welfare spending, emphasizing the need of addressing these challenges in order to improve maternal and child health outcomes in the Local Health Departments (LHD). The identified determinants of adverse teenage pregnancy outcomes were age group, physical exercise, drug use, premature delivery, smoking, followed by medical prescription, residence and feeding properly. These findings point out the importance of educating and empowering teenage girls to help them make the right decisions. Also, addressing these adverse outcomes of teenage pregnancy requires a multidimensional approach that encompasses healthcare services, education, and social support to reduce the risk faced by teenage mothers and their children.

### What is known about this topic

- Teenage pregnancy is associated with various adverse outcomes, including increased rates of cesarean delivery, preterm birth, and low birth weight;
- Previous studies indicate that socioeconomic factors, maternal age, and health behaviors significantly influence pregnancy outcomes in adolescents;
- High neonatal mortality rates are observed among teenage mothers, often linked to inadequate prenatal care and poor nutritional status.

### What this study adds

- This study found a 34.8% prevalence of adverse outcomes related to teenage pregnancies, highlighting significant health risks for this population;
- Determinants of cesarean delivery included drug use, premature delivery, and lack of physical exercise among teenage mothers;
- Factors influencing low birth weight and neonatal mortality were identified, including age group, adherence to medical prescriptions, and proper nutrition during pregnancy.
